# Does RAIM with Correct Exclusion Produce Unbiased Positions?

**DOI:** 10.3390/s17071508

**Published:** 2017-06-26

**Authors:** Peter J. G. Teunissen, Davide Imparato, Christian C. J. M. Tiberius

**Affiliations:** 1 GNSS Research Centre, Curtin University of Technology, 6845 Perth, Australia; davide.imparato@curtin.edu.au; 2 Department of Geoscience and Remote Sensing, Delft University of Technology, 2628 CN Delft, The Netherlands; c.c.j.m.tiberius@tudelft.nl

**Keywords:** Receiver Autonomous Integrity Monitoring (RAIM), best linear unbiased estimation (BLUE), statistical hypothesis Testing, missed detection (MD), correct detection (CD), correct identification (CI), level of significance, bias, Global Navigation Satellite System (GNSS)

## Abstract

As the navigation solution of exclusion-based RAIM follows from a combination of least-squares estimation and a statistically based exclusion-process, the computation of the integrity of the navigation solution has to take the propagated uncertainty of the combined estimation-testing procedure into account. In this contribution, we analyse, theoretically as well as empirically, the effect that this combination has on the first statistical moment, i.e., the mean, of the computed navigation solution. It will be shown, although statistical testing is intended to remove biases from the data, that biases will *always* remain under the alternative hypothesis, even when the correct alternative hypothesis is properly identified. The a posteriori exclusion of a biased satellite range from the position solution will therefore never remove the bias in the position solution completely.

## 1. Introduction

Statistical inference principles as estimation and testing play a fundamental role in the broad spectrum of navigation applications. Estimation is then usually aimed at finding unbiased estimators having the best possible precision, while testing is used to safeguard against incorrect modeling and its consequences. In the safety-critical navigation application of aviation, the concept of Receiver Autonomous Integrity Monitoring (RAIM) was specifically developed to safeguard the navigation integrity by means of self-contained fault detection at the GNSS navigation receiver [1,2]. With RAIM, the use of statistical hypothesis testing is commonplace. One may test the validity of the assumed working hypothesis by means of a chi-square distributed sum-of-squared-residuals [3,4], or more specifically, test the working hypothesis against specified alternatives (e.g., single satellite faults) through Gaussian distributed test-statistics [5,6]. Depending on the parametrization of the underlying model, many different implementations of these test-statistics exist [7,8,9,10,11]. Next to aviation, RAIM-testing finds its application also in a broad range of other applications (see, e.g., [12,13,14,15,16,17,18,19]).

In RAIM, estimation and testing are combined, which results in an overall estimator that is more complex than when one would treat estimation and testing separately. That is, the navigation solution of exclusion-based RAIM is the result of a combination of least-squares estimation and a statistical pseudorange ’inclusion–exclusion’ process. This combination of estimation and testing implies that one cannot assign the properties of the estimators under the different hypotheses to the actual estimator computed. In RAIM without exclusion, this is not an issue as one is then only working with one single estimator, namely with the one computed under the null hypothesis in case of acceptance, while in case of rejection, no estimator is computed as the solution is then said to be unavailable. Hence, in RAIM without exclusion, the distributional properties of the estimator are known, which can then directly be used to compute and evaluate the probability of hazardous misleading information. This situation changes however when exclusion is included in the process. In that case, one is not dealing with one single estimator, but actually with a combination of multiple estimators, one for each hypothesis specified. Hence, to obtain a probabilistic description of this combination, one will have to take the uncertainty of the combined estimation-testing procedure into account and perform the propagation of uncertainty accordingly. In this contribution, we explain the mechanism of this interplay and study its effect on the first moment of the distribution. We analyse, theoretically as well as empirically, how the mean of the computed estimator is affected by this interplay.

This contribution is organized as follows. We start in Section 2 with a brief review of the necessary estimation and testing results of linear model theory. We then make the case in Section 3 that, although estimation and testing are often treated separately and independently, in actual practice, they are usually combined, thus the navigation solution of exclusion-based RAIM is the product of a least-squares estimation that is applied to pseudoranges that already underwent a statistical ’inclusion–exclusion’ process. In Section 3, we identify the consequences of the combined estimation-testing procedure has for the distribution of the estimators involved and in particular we show that the estimators remain biased even if the correct alternative hypothesis has been identified. These results are then generalized in Section 4 for the case of having multiple alternative hypotheses, for which the events of correct detection and correct identification then need to be separated out. In Section 5, we apply the theoretical results and demonstrate the presence of said biases in the single-receiver pseudorange GNSS positioning results of exclusion-based RAIM. This shows that the a posteriori exclusion of a biased satellite range will never remove the bias in the position solution completely. The size of such remaining biases depends on the strength of the underlying model, the chosen false alarm rate, and the size and type of the actual input bias. We show how these biases can be computed and that an increased model strength and larger levels of significance allow for a reduction of the bias. The contribution is finally concluded with a summary in Section 6.

## 2. Estimation and Testing

In this section, we briefly review some necessary estimation and testing results of linear model theory.

### 2.1. Estimation

Consider the linear observation model

(1) $$H_{0}:\;\;E\left(y\right)=Ax\;,\;D\left(y\right)=Q_{yy},$$

with E(.) the expectation operator, $y\in R^{m}$ the normally distributed random vector of observables, $A\in R^{m\times n}$ the given design matrix of rank *n*, and $x\in R^{n}$ the to-be-estimated unknown parameter vector, D(.) the dispersion operator and $Q_{yy}\in R^{m\times m}$ the given positive-definite variance matrix of *y*. The linear model (1) will be referred to as our null-hypothesis $H_{0}$.

Under $H_{0}$, the best linear unbiased estimator (BLUE) of *x* is given as

(2) $${\hat{x}}_{0}=A^{+}y,$$

with least-squares (LS) inverse $A^{+}=Q_{{\hat{x}}_{0}{\hat{x}}_{0}}A^{T}Q_{yy}^{-1}$, in which $Q_{{\hat{x}}_{0}{\hat{x}}_{0}}=D\left({\hat{x}}_{0}\right)={\left(A^{T}Q_{yy}^{-1}A\right)}^{-1}$ is the dispersion or variance matrix of ${\hat{x}}_{0}$.

As the BLUE’s property of ${\hat{x}}_{0}$ depends on the validity of $H_{0}$, it is important that one has sufficient confidence in the assumptions underlying the null-hypothesis. Although every part of the null-hypothesis can be wrong of course, we assume here that if a misspecification occurred, it is confined to an under-parametrization of the mean of *y*, in which case

(3) $$H_{a}:\;\;E\left(y\right)=Ax+Cb\;,\;D\left(y\right)=Q_{yy}$$

for some vector $b_{y}=Cb$. Experience has shown that these types of misspecifications are by and large the most common errors that occur when formulating the model. Note that formulation (3) is general in the sense that, through the choice of matrix *C*, it allows one to capture any type of additive mismodelling in the observation equations.Through $b_{y}=Cb$, one may model, for instance, the presence of one or more blunders (outliers) in the data, cycle-slips in phase data, satellite failures, antenna-height errors, erroneous neglecting of atmospheric delays, or any other systematic effect that one failed to take into account under $H_{0}$.

In the following, we assume matrix $\left[A,C\right]\in R^{m\times (n+q)}$ to be known of rank n+q and the parameter vector $b\in R^{q}$ to be unknown. The linear model (3) will be referred to as the alternative-hypothesis $H_{a}$.

Under $H_{a}$, the BLUE of *x* is given as

(4) $${\hat{x}}_{a}={\bar{A}}^{+}y,$$

with LS-inverse ${\bar{A}}^{+}=Q_{{\hat{x}}_{a}{\hat{x}}_{a}}{\bar{A}}^{T}Q_{yy}^{-1}$, $\bar{A}=P_{C}^{⊥}A$, $P_{C}^{⊥}=I_{m}-C{\left(C^{T}Q_{yy}^{-1}C\right)}^{-1}C^{T}Q_{yy}^{-1}$, and $Q_{{\hat{x}}_{a}{\hat{x}}_{a}}=D\left({\hat{x}}_{a}\right)={\left({\bar{A}}^{T}Q_{yy}^{-1}\bar{A}\right)}^{-1}$. As this BLUE is based on a model with more parameters, its precision will never be better than that of ${\hat{x}}_{0}$, i.e., $D\left({\hat{x}}_{0}\right)\leq D\left({\hat{x}}_{a}\right)$.

### 2.2. Testing

The estimation of *x* would not pose a problem if we would know which of the two models would be true. In the case of $H_{0}$, we would use ${\hat{x}}_{0}$ to estimate *x*, but if we would know that $H_{a}$ is true, then we would use ${\hat{x}}_{a}$ instead. Using the estimator ${\hat{x}}_{0}$ when knowing that $H_{a}$ is true should be avoided, as this would result in a biased solution, since

(5) $$E\left({\hat{x}}_{0}|H_{a}\right)=x+A^{+}Cb.$$

The problem in practice of course is that we do not know which of the models are true. Even if we have taken the utmost care in formulating a model which we believe to be true, misspecifications could still be present, thus invalidating the model. Methods of statistical testing have therefore been developed that allow us to decide with some confidence which of the models to work with. In the case of the above $H_{0}$ and $H_{a}$, it seems reasonable to decide in favour of $H_{0}$ if the BLUE of *b* can be considered ’insignificant’. With the BLUE of *b* under $H_{a}$ given as

(6) $$\hat{b}={\bar{C}}^{+}y,$$

with LS-inverse ${\bar{C}}^{+}={\left({\bar{C}}^{T}Q_{yy}^{-1}\bar{C}\right)}^{-1}{\bar{C}}^{T}Q_{yy}^{-1}$, $\bar{C}=P_{A}^{⊥}C$ and variance matrix $Q_{\hat{b}\hat{b}}={\left({\bar{C}}^{T}Q_{yy}^{-1}\bar{C}\right)}^{-1}$, the decision in favour of $H_{0}$ is therefore taken when $\hat{b}$ lies in the acceptance region A,

(7) $$\hat{b}\in A=\{b\in R^{q}{\;|\;||b||}_{Q_{\hat{b}\hat{b}}}^{2}\leq \chi_{\alpha }^{2}\left(q,0\right)\}$$

with ${\left||.|\right|}_{Q_{\hat{b}\hat{b}}}^{2}={\left(.\right)}^{T}Q_{\hat{b}\hat{b}}^{-1}\left(.\right)$ and $\chi_{\alpha }^{2}\left(q,0\right)$ the critical value computed from the central chi-square distribution with *q* degrees of freedom and chosen level of significance α. This test is known to be a uniformly most powerful invariant (UMPI) test for testing $H_{0}$ against $H_{a}$ [7,20]. Note that test (7) should not be confused with the sum-of-squared-residuals test. The two are only the same in the special case that the least-squares residual under the alternative becomes identically zero (cf. (8)). To give further meaning to the statistic $\left|\right|\hat{b}{\left|\right|}_{Q_{\hat{b}\hat{b}}}^{2}$, we note that it can be written in different ways ([7], p. 79), two of which are

(8) $$\begin{array}{ccc} \left|\right|\hat{b}{\left|\right|}_{Q_{\hat{b}\hat{b}}}^{2} & = & \left|\right|{\hat{y}}_{0}-{\hat{y}}_{a}{\left|\right|}_{Q_{yy}}^{2}\\ \; & = & \left|\right|{\hat{e}}_{0}{\left|\right|}_{Q_{yy}}^{2}-\left|\right|{\hat{e}}_{a}{\left|\right|}_{Q_{yy}}^{2}, \end{array}$$

with ${\hat{y}}_{0}=A{\hat{x}}_{0}$, ${\hat{y}}_{a}=A{\hat{x}}_{a}+C\hat{b}$, ${\hat{e}}_{0}=y-{\hat{y}}_{0}$, and ${\hat{e}}_{a}=y-{\hat{y}}_{a}$. The first equality of (8) states that the statistic is equal to the squared norm of the solution separation in the observation domain. Thus, if this solution separation is small enough, one has no reason for rejecting $H_{0}$. The second equality of (8) states that the statistic is also equal to the difference of the squared norm residuals under $H_{0}$ and $H_{a}$. Thus, again, if this difference is small enough, the decision is that there is no reason for mistrusting $H_{0}$. Although we will be using the first expression of (8) for the test statistic, linkage to the other two will be made when we discuss multiple hypothesis testing in Section 4.

If the outcome of testing is to reject $H_{0}$ in favour of $H_{a}$, then not ${\hat{x}}_{0}$, but ${\hat{x}}_{a}$ is provided as the estimator for *x*. The three estimators, ${\hat{x}}_{0}$ (cf. (2)), ${\hat{x}}_{a}$ (cf. (4)) and $\hat{b}$ (cf. (6)) are related as

(9) $${\hat{x}}_{a}={\hat{x}}_{0}-A^{+}C\hat{b}.$$

Thus, if $H_{0}$ is rejected in favour of $H_{a}$, then $A^{+}C\hat{b}$ is the correction, which is aimed at removing the bias $A^{+}Cb$ (cf. (5)) from ${\hat{x}}_{0}$. Whether or not this is actually achieved is discussed in the following sections.

## 3. Estimation Bias Due to Testing

### 3.1. The Estimator Revisited

**Figure 1:**
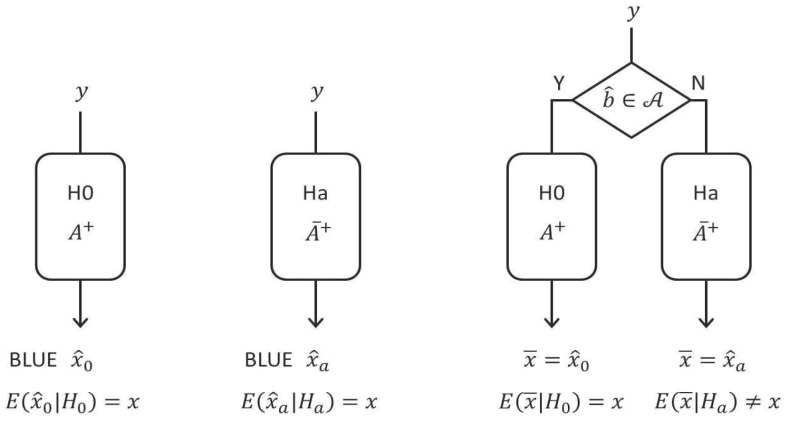
(left) $H_{0}$ known: ${\hat{x}}_{0}$ is BLUE of *x*; (middle) $H_{a}$ known: ${\hat{x}}_{a}$ is BLUE of *x*; (right) $H_{0}$ and $H_{a}$ selected under chance: $\bar{x}$ not a BLUE of *x* (cf. (10), (12) and (13)).

As mentioned above, estimation and testing are combined, so one cannot assign the properties of ${\hat{x}}_{0}$ or ${\hat{x}}_{a}$ to the actual estimator that is computed. That is, the actual estimator that is produced is not ${\hat{x}}_{0}$ nor ${\hat{x}}_{a}$, but in fact (see Figure 1).

(10) $$\bar{x}=\left\{\begin{array}{cc} {\hat{x}}_{0}, & \mathrm{if}\;\;\hat{b}\in A,\\ {\hat{x}}_{a}, & \mathrm{if}\;\;\hat{b}\notin A. \end{array}\right.$$

Hence, it is the quality of $\bar{x}$, rather than that of ${\hat{x}}_{0}$ or ${\hat{x}}_{a}$, that determines the quality of the produced results. Since ideally the goal of testing is to be able to have the bias $A^{+}Cb$ removed from ${\hat{x}}_{0}$ when $H_{a}$ is true (cf. (5)), it is relevant to know what the mean of the actual estimator $\bar{x}$ is. By making use of the relation (9), the expectation of $\bar{x}$ can be determined as

(11) $$\begin{array}{ccc} E(\bar{x}|H_{0}) & = & x-A^{+}C\int_{\notin A}\beta p_{\hat{b}}\left(\beta |H_{0}\right)d\beta ,\\ E(\bar{x}|H_{a}) & = & x+A^{+}C\int_{\in A}\beta p_{\hat{b}}\left(\beta |H_{a}\right)d\beta , \end{array}$$

with $p_{\hat{b}}\left(\beta |H_{0}\right)$ and $p_{\hat{b}}\left(\beta |H_{a}\right)$ being the probability density function (PDF) of $\hat{b}$ under resp. $H_{0}$ and $H_{a}$.

The result (11) shows that the estimator $\bar{x}$ is biased in general. This in contrast to ${\hat{x}}_{0}$ under $H_{0}$ and ${\hat{x}}_{a}$ under $H_{a}$. The cause for the presence of these biases is the *nonlinearity* involved in the mapping of (10). Thus, although ${\hat{x}}_{0}$ and ${\hat{x}}_{a}$ are both individually linear functions of *y*, the actually produced estimator $\bar{x}$ is not. It is this nonlinearity that prohibits the unbiasedness of ${\hat{x}}_{0}$ and ${\hat{x}}_{a}$, under, respectively, $H_{0}$ and $H_{a}$, to be passed on to $\bar{x}$.

Although (11) indicates that $\bar{x}$ is generally biased under both $H_{0}$ and $H_{a}$, we have in our case $\int_{\notin A}\beta p_{\hat{b}}\left(\beta |H_{0}\right)d\beta =0$, due to the *symmetry* with respect to the origin of both the acceptance region A and the PDF $p_{\hat{b}}\left(\beta |H_{0}\right)$. Hence, in our case, the estimator $\bar{x}$ is fortunately always unbiased under $H_{0}$:

(12) $$E(\bar{x}|H_{0})=x.$$

This is not true, however, for $\bar{x}$ under $H_{a}$. We have

(13) $$E\left(\bar{x}|H_{a}\right)=x+b_{\bar{x}},$$

with the bias given as

(14) $$b_{\bar{x}}=A^{+}Cb_{A}\;\;\mathrm{with}\;\;b_{A}=\int_{\in A}\beta p_{\hat{b}}\left(\beta |H_{a}\right)d\beta .$$

This shows that the bias in $\bar{x}$ is driven by the vector $b_{A}$ and its propagation into the parameter space. The vector $b_{A}$ itself is governed by the acceptance region A and through the PDF $p_{\hat{b}}\left(\beta |H_{a}\right)$, by the actual bias *b* and the precision with which it can be estimated, $Q_{\hat{b}\hat{b}}$. Generally, $p_{\hat{b}}\left(\beta |H_{a}\right)$ is not symmetric over region A.

To see the effect testing has, one can compare the testing-induced bias (14), with the bias one otherwise would have when using ${\hat{x}}_{0}$ under $H_{a}$ (cf. (5)),

(15) $$b_{{\hat{x}}_{0}}=E\left({\hat{x}}_{0}-x|H_{a}\right)=A^{+}Cb.$$

It follows from comparing (14) with (15), since $b=E\left(\hat{b}|H_{a}\right)=\int_{R^{q}}\beta p_{\hat{b}}\left(\beta |H_{a}\right)d\beta$, that through testing, it is the component of this integral over the acceptance region A that is retained. We thus have $b_{\bar{x}}=b_{{\hat{x}}_{0}}$ if $A=R^{q}$, which corresponds to the case of always accepting $H_{0}$.

A summary overview of the means of the random vectors ${\hat{x}}_{0}$, ${\hat{x}}_{a}$ and $\bar{x}$ is given in Table 1. Note that over-parametrization, i.e., using model (3) rather than (1), delivers unbiased results: $E({\hat{x}}_{a}|H_{0})=x$.

### 3.2. The One-Dimensional Case

**Figure 2:**
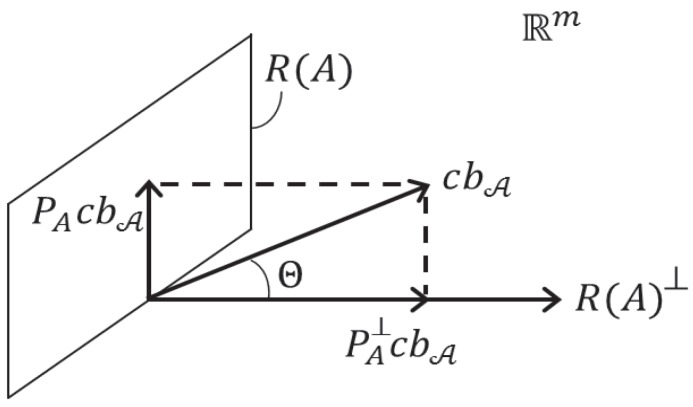
Orthogonal decomposition of $cb_{A}$ into range space of *A* and its orthogonal complement: $cb_{A}=\left(P_{A}c+P_{A}^{⊥}c\right)b_{A}$ [7].

As mentioned above, the testing induced-bias $b_{\bar{x}}$ is driven by $b_{A}$ and its propagation into the parameter space. To describe its significance, we will work with the dimensionless bias-to-noise ratio (BNR) $\left|\right|b_{\bar{x}}{\left|\right|}_{Q_{{\hat{x}}_{0}{\hat{x}}_{0}}}$ and study its behaviour for the one-dimensional case. If q=1, then matrix *C* becomes a vector, C=c, and *b* becomes a scalar. For this case the BNR works out as

(16) $$\left|\right|b_{\bar{x}}{\left|\right|}_{Q_{{\hat{x}}_{0}{\hat{x}}_{0}}}=\frac{|b_{A}|}{\sigma_{\hat{b}}}\tan\theta ,$$

with θ being the angle that vector *c* makes with the range space of the orthogonal complement of *A*, i.e., $\tan\theta =\left|\right|P_{A}{c||}_{Q_{yy}}/\left|\right|P_{A}^{⊥}c{\left|\right|}_{Q_{yy}}$ [7], p. 111. Here, $P_{A}=AA^{+}$ and $P_{A}^{⊥}=I_{m}-AA^{+}$. In the decomposition (16), $|b_{A}|/\sigma_{\hat{b}}$ describes the significance of $b_{A}$, while tanθ shows how it gets propagated into the parameter space (Figure 2).

There are two cases for which $b_{A}$ will be ’small’. It will be small when the PDF $p_{\hat{b}}\left(\beta |H_{a}\right)$ has only a small portion of its probability mass over A, and it will be small when it differs only a little from the PDF under $H_{0}$. To quantify this behaviour, we make use of the one-dimensional integral (cf. (14))

(17) $$b_{A}=\frac{1}{\sqrt{2\pi }\sigma_{\hat{b}}}\int_{-\chi_{\alpha }\left(1,0\right)\sigma_{\hat{b}}}^{\chi_{\alpha }\left(1,0\right)\sigma_{\hat{b}}}\beta \exp\left\{-\frac{1}{2}{\left(\frac{\beta -b}{\sigma_{\hat{b}}}\right)}^{2}\right\}d\beta ,$$

from which it can be worked out that, with $\chi_{\alpha }\left(1,0\right)=\sqrt{\chi_{\alpha }^{2}\left(1,0\right)}$,

(18) $$\frac{b_{A}}{\sigma_{\hat{b}}}=F\left(\chi_{\alpha }\left(1,0\right)\right)-F\left(-\chi_{\alpha }\left(1,0\right)\right),$$

in which $F\left(x\right)=\phi \left(\frac{b}{\sigma_{\hat{b}}}+x\right)+\frac{b}{\sigma_{\hat{b}}}\Phi \left(\frac{b}{\sigma_{\hat{b}}}+x\right)$ with $\phi \left(x\right)=\frac{1}{\sqrt{2\pi }}\exp\left\{-\frac{1}{2}x^{2}\right\}$ and $\Phi \left(x\right)=\int_{-\infty }^{x}\phi \left(v\right)dv$.

**Figure 3:**
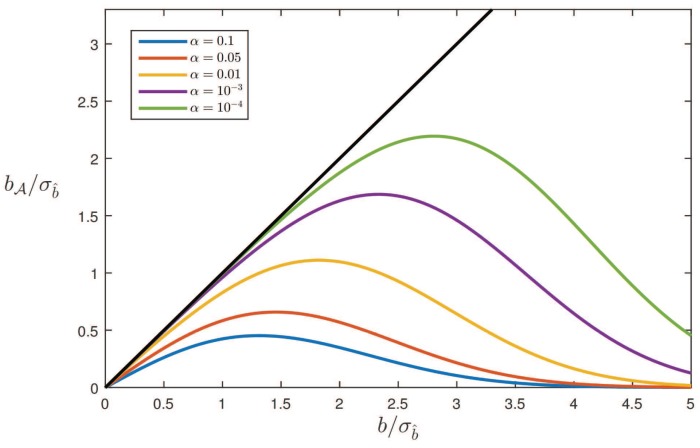
Bias $b_{A}/\sigma_{\hat{b}}$ as function of $b/\sigma_{\hat{b}}$ for different values of α.

Figure 3 shows $b_{A}/\sigma_{\hat{b}}$ as a function of $b/\sigma_{\hat{b}}$ for different values of α (here, and in the following, we consider b≥0). The straight line in the figure describes the bias one would have in case no testing would be performed (i.e., A=R). As $b_{A}\leq b$ for every value of *b*, the figure clearly shows the benefit of testing: the bias that remains after testing is always smaller than the original bias. Note that this benefit, i.e., the difference between *b* and $b_{A}$, only kicks in after the bias *b* has become large enough. The difference is small, when *b* is small, and it gets larger for larger *b*, with $b_{A}$ approaching zero in the limit. Also note that for smaller levels of significance α, the difference between *b* and $b_{A}$ stays small for a larger range of *b*-values. This is understandable as a smaller α corresponds with a larger acceptance interval A, as a consequence of which one would have for a larger range of *b*-values an outcome of testing that does not differ from the no-testing scenario.

### 3.3. The Conditional Mean

We have shown that the combination of estimation and testing always produces a biased estimator under $H_{a}$, that is, a fraction of the original bias *b* will always be passed on to the estimator $\bar{x}$ when $H_{a}$ is true. However, the mean considered so far is an unconditional mean, i.e., one that does not take the outcome of testing into account. We therefore now consider the mean of $\bar{x}$ under the condition of either incorrect acceptance or correct rejection of $H_{0}$, i.e., under the condition that $\hat{b}\in A$ or $\hat{b}\notin A$ while $H_{a}$ is true.

It follows from (10) and (15) and the independence of $\hat{b}$ and ${\hat{x}}_{0}$ that

(19) $$E\left(\bar{x}|\hat{b}\in A,H_{a}\right)=E\left({\hat{x}}_{0}|H_{a}\right)=x+b_{{\hat{x}}_{0}}.$$

Thus, in case of wrongful acceptance, the bias of $\bar{x}$ is that of ${\hat{x}}_{0}$.

We now consider the case of correct rejection, i.e., $\hat{b}\notin A$ while $H_{a}$ is true. In that case, we have for the mean

(20) $$E\left(\bar{x}|\hat{b}\notin A,H_{a}\right)=E\left({\hat{x}}_{a}|\hat{b}\notin A,H_{a}\right)=x+b_{\bar{x}|\hat{b}\notin A},$$

with

(21) $$\begin{array}{ccc} b_{\bar{x}|\hat{b}\notin A} & = & A^{+}C\left(b_{A}-\frac{P(\hat{b}\in A|H_{a})}{P(\hat{b}\notin A|H_{a})}\left(b-b_{A}\right)\right)\\ \; & = & A^{+}C\left(b-\frac{b-b_{A}}{1-P(\hat{b}\in A|H_{a})}\right)=A^{+}Cb_{A|\hat{b}\notin A}. \end{array}$$

This result is obtained as follows. From taking the conditional expectation of (9) and noting that ${\hat{x}}_{0}$ and $\hat{b}$ are independent, we obtain $E\left({\hat{x}}_{a}|\hat{b}\notin A,H_{a}\right)=E\left({\hat{x}}_{0}|H_{a}\right)-A^{+}CE\left(\hat{b}|\hat{b}\notin A,H_{a}\right)$, from which the result follows by using (15). Note how the two expressions of (21) show how the bias compares to that of $\bar{x}$ (cf. (14)) and to that of ${\hat{x}}_{0}$ (cf. (15)). Also note that the above provided general formula (21) allows one to evaluate the parameter effect $b_{\bar{x}|\hat{b}\notin A}$ for any size and any type of bias. A summary overview of the conditional means of $\bar{x}$ is given in Table 2.

**Figure 4:**
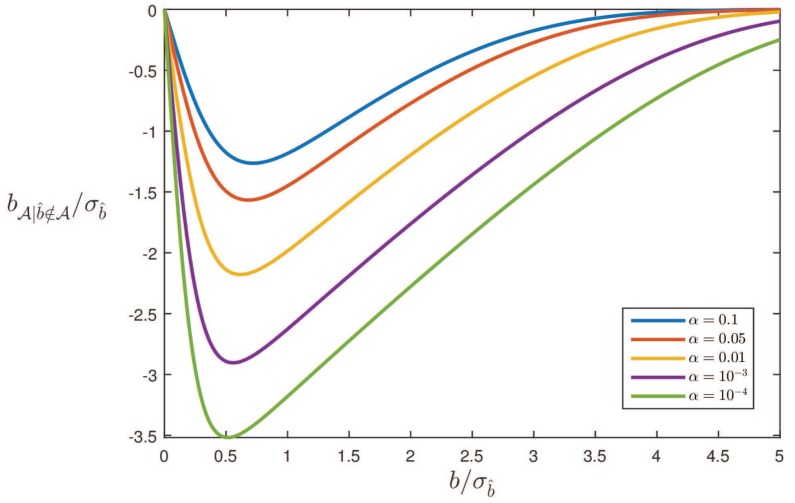
Bias, for correct rejection of $H_{0}$, $b_{A|\hat{b}\notin A}/\sigma_{\hat{b}}$ as function of $b/\sigma_{\hat{b}}$ for different values of α, where $b_{A|\hat{b}\notin A}=b-\left(b-b_{A}\right)/\left(1-P\left(\hat{b}\in A|H_{a}\right)\right)$.

Figure 4 shows $b_{A|\hat{b}\notin A}/\sigma_{\hat{b}}$ as a function of $b/\sigma_{\hat{b}}$ for different values of α. Note, since $b_{A|\hat{b}\notin A}=b-E\left(\hat{b}|\hat{b}\notin A,H_{a}\right)\leq 0$, that the testing induced bias-correction overcompensates for the bias *b*. This can be explained by the shape of the conditional PDF $p_{\hat{b}|\hat{b}\notin A}\left(x|H_{a}\right)$. As the probability mass of the unconditional PDF over the origin centred acceptance interval A has been distributed over its complement, the conditional PDF has more probability mass on the right side of *b* than on the left side of *b*, as a consequence of which its mean value will be *larger* than *b*. Furthermore, since the probability mass that gets distributed over the complement of A increases for a smaller α, the overcompensation also gets larger for smaller α.

The results shown in the Figure 3 and Figure 4 are linked by the probability of correct detection, i.e., the power of the test. It follows from (21) that

(22) $$\left[b-b_{A}\right]=P\left(\hat{b}\notin A|H_{a}\right)\left[b-b_{A|\hat{b}\notin A}\right].$$

Hence, particularly when the probability of correct detection is small, there will be large differences between $b_{A}$ and $b_{A|\hat{b}\notin A}$. Furthermore, since the probability of detection gets smaller for smaller α, in particular for not too large *b*, also the differences between $b_{A}$ and $b_{A|\hat{b}\notin A}$ get larger for smaller α.

Note that where in the unconditional case the remaining bias $b_{A}$ is smaller than *b* in absolute value (see Figure 3), this is *not* the case with the conditional bias $b_{A|\hat{b}\notin A}$ (see Figure 4). In the conditional case of correct detection, the size of the bias remaining after testing can even be larger than the original bias. This may come as a surprise, but is a direct consequence of the fact that in this conditional case, the samples of $\hat{b}$ that come from the distribution under $H_{a}$, but lie in the acceptance region A, are not considered. If for one’s application, the size of the bias $b_{A|\hat{b}\notin A}$ is considered too large, one has the following remedy available: one can either design a stronger model, thus giving a smaller $\sigma_{\hat{b}}$, or one can decide to work with a larger level of significance α, thereby accepting more false rejections of $H_{0}$.

## 4. Multiple Alternative Hypotheses

### 4.1. Test Procedure

Up to now, we have been working with only one single alternative hypothesis $H_{a}$. In actual practice, however, one typically works with many more such hypotheses, namely with one for every misspecification that one believes has sufficient potential of occurrence. Hence, one will then have a set of, say *m*, alternative hypotheses,

(23) $$H_{a_{i}}:\;\;E\left(y\right)=Ax+C_{i}b_{i}\;,\;D\left(y\right)=Q_{yy},$$

in which each $C_{i}b_{i}$, i=1,…,m, is assumed to take care of one of the potential misspecifications. Also in this case, biases will remain in the computed results of the combined estimation and testing process [21]. We will show this by means of one of the most commonly used multiple hypothesis testing problems, namely the screening of observations for possible outliers. As we consider one outlier at a time, matrix $C_{i}=c_{i}$ becomes the canonical unit vector having 1 as its *i*th entry, with $b_{i}$ being the scalar outlier and *i* indicating the potentially outlier-affected observation. In total, *m* tests are carried out. The applied decision rule is then to accept the null hypothesis, unless

(24) $$\max_{i\in \{1,\ldots ,m\}}\frac{|{\hat{b}}_{i}|}{k_{i}\sigma_{{\hat{b}}_{i}}}>1\;\;\;\mathrm{with}\;\;k_{i}=\chi_{\alpha }\left(1,0\right),$$

in which case the corresponding hypothesis is selected. Once a hypothesis, say $H_{a_{j}}$, is selected, the parameter vector is estimated as

(25) $${\hat{x}}_{a_{j}}={\bar{A}}_{\left(j\right)}^{+}y={\hat{x}}_{0}-A^{+}c_{j}{\hat{b}}_{j},$$

in which ${\bar{A}}_{\left(j\right)}=P_{c_{j}}^{⊥}A$. Note that in case of uncorrelated observations, i.e., $Q_{yy}=\mathrm{diag}\left(\sigma_{1}^{2},\ldots ,\sigma_{m}^{2}\right)$, the adapted design matrix ${\bar{A}}_{\left(j\right)}=P_{c_{j}}^{⊥}A$ is the original design matrix with its jth row replaced by zeros. Hence, ${\hat{x}}_{a_{j}}$ is then the estimator with the jth observable *excluded*.

Note that in (24), no preference is given to the contributions of any of the *m* individual statistics $|{\hat{b}}_{i}|/\sigma_{{\hat{b}}_{i}}$, i.e., $k_{i}$ is the same for all *i*. Although this is a common usage that we will follow in this contribution, it is not necessary, as one could also weigh the contributions of the individual statistics in dependence of their importance for the computed results, i.e., by testing more stringently for biases $c_{i}b_{i}$ that have a larger impact on the parameters of interest.

### 4.2. Alternative Statistics

With reference to our earlier alternative expressions of the test statistic (cf. (8)), we note that instead of maximizing $|{\hat{b}}_{i}|/\sigma_{{\hat{b}}_{i}}$ to identify the alternative hypothesis, one may also do this by finding the minimizing $\left|\right|{\hat{e}}_{i}{\left|\right|}_{Q_{yy}}^{2}$ [2] and then use the difference $\left|\right|{\hat{e}}_{0}{\left|\right|}_{Q_{yy}}^{2}-\left|\right|{\hat{e}}_{i}{\left|\right|}_{Q_{yy}}^{2}$ as a test statistic.

**Figure 5:**
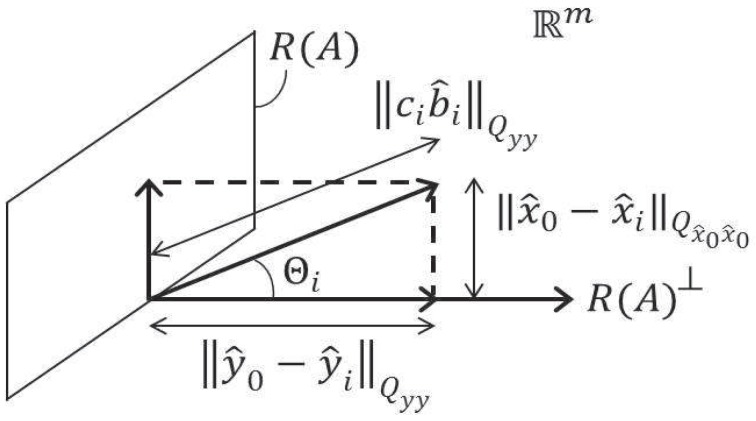
Orthogonal decomposition of solution separation statistics: $\left|\right|c_{i}{\hat{b}}_{i}{\left|\right|}_{Q_{yy}}^{2}=\left|\right|{\hat{y}}_{0}-{\hat{y}}_{i}{\left|\right|}_{Q_{yy}}^{2}+\left|\right|{\hat{x}}_{0}-{\hat{x}}_{i}{\left|\right|}_{Q_{{\hat{x}}_{0}{\hat{x}}_{0}}}^{2}$.

Similarly, we noted (cf. (8)) that one may use the solution separation statistic in the observation domain $\left|\right|{\hat{y}}_{0}-{\hat{y}}_{i}{\left|\right|}_{Q_{yy}}^{2}=\left|\right|P_{A}^{⊥}C_{i}{\hat{b}}_{i}{\left|\right|}_{Q_{yy}}^{2}$, instead of $\left|\right|\hat{b}{\left|\right|}_{Q_{\hat{b}\hat{b}}}^{2}$. By using the Pythagorean rule (see Figure 5), we therefore have in the scalar case, when $C_{i}=c_{i}$, for the corresponding solution separation statistic in the *parameter* domain the relation

(26) $$\left|\right|{\hat{x}}_{0}-{\hat{x}}_{i}{\left|\right|}_{Q_{{\hat{x}}_{0}{\hat{x}}_{0}}}^{2}=\left|\right|{\hat{y}}_{0}-{\hat{y}}_{i}{\left|\right|}_{Q_{yy}}^{2}\tan^{2}\theta_{i},$$

with $\tan^{2}\theta_{i}=\left|\right|P_{A}c_{i}{\left|\right|}_{Q_{yy}}^{2}/\left|\right|P_{A}^{⊥}c_{i}{\left|\right|}_{Q_{yy}}^{2}$. Thus, if one would like to perform the test procedure (24) using (26), then the maximum of $\left|\right|{\hat{x}}_{0}-{\hat{x}}_{i}{\left|\right|}_{Q_{{\hat{x}}_{0}{\hat{x}}_{0}}}/(k_{i}|\tan\theta_{i}\left|\right)$ is sought for. Likewise, if $k_{i}$ in (24) would be set as $k_{i}=k/\left|\tan\theta_{i}\right|$, with *k* chosen such that the overall false-alarm rate remains the same, one would be using $\max_{i\in \{1,\ldots ,m\}}\left|\right|{\hat{x}}_{0}-{\hat{x}}_{i}{\left|\right|}_{Q_{{\hat{x}}_{0}{\hat{x}}_{0}}}>k$ as the decision rule. In this case, one would thus be testing less stringently for model biases $c_{i}b_{i}$ that have a small influence on the parameter solution, i.e., for which the $\theta_{i}$ are small.

Finally, note that in the scalar case, when $C_{i}=c_{i}$, one may also use any linear function of the parameter solution separation as a test statistic. As it follows from (25) that $f^{T}\left({\hat{x}}_{0}-{\hat{x}}_{i}\right)=\left(f^{T}A^{+}c_{i}\right){\hat{b}}_{i}$, we have

(27) $$\frac{|{\hat{b}}_{i}|}{\sigma_{{\hat{b}}_{i}}}=\frac{|f^{T}\left({\hat{x}}_{0}-{\hat{x}}_{i}\right)|}{\sigma_{f^{T}\left({\hat{x}}_{0}-{\hat{x}}_{i}\right)}},$$

for every nonzero $f\in R^{n}$. Hence, the choice of *f* is in this case of no consequence for the outcome of testing.

### 4.3. Biases

We will now examine how the combined estimation and multiple testing affects the mean of the computed parameters. As we now have with (3) more than one alternative hypothesis, the correct detection of a mismodeled $H_{0}$ is not the same anymore as the correct identification of an alternative hypothesis. For correct detection (CD), assuming $H_{a_{1}}$ to be true, the occurrence $|{\hat{b}}_{j}|/\sigma_{{\hat{b}}_{j}}>\chi_{\alpha }\left(1,0\right)$, with $j=\arg\max_{i}\left(\right|{\hat{b}}_{i}\left|/\sigma_{{\hat{b}}_{i}}\right)$, needs to happen for some j∈{1,…,m}, while for correct identification (CI), the occurrence needs to happen for j=1 only. We therefore now consider the following three biases under $H_{a_{1}}$: the unconditional bias $b_{\bar{x}}=E\left(\bar{x}-x|H_{a_{1}}\right)$, the bias conditioned on correct detection, $b_{\bar{x}|\mathrm{CD}}=E\left(\bar{x}-x|\mathrm{CD},H_{a_{1}}\right)$, and the bias conditioned on correct identification, $b_{\bar{x}|\mathrm{CI}}=E\left(\bar{x}-x|\mathrm{CI},H_{a_{1}}\right)$. These three biases will be first illustrated by means of a simple example.

### Example: Averaging

In this example, the data are generated from a model of the form

(28) $$H_{a_{1}}:E\left(y\right)=Ax+c_{1}b\;,\;D\left(y\right)=I_{m},$$

in which

(29) $$A={\left[1,\ldots ,1\right]}^{T}\;\;\mathrm{and}\;\;c_{1}={\left[1,0,\ldots ,0\right]}^{T}.$$

**Figure 6:**
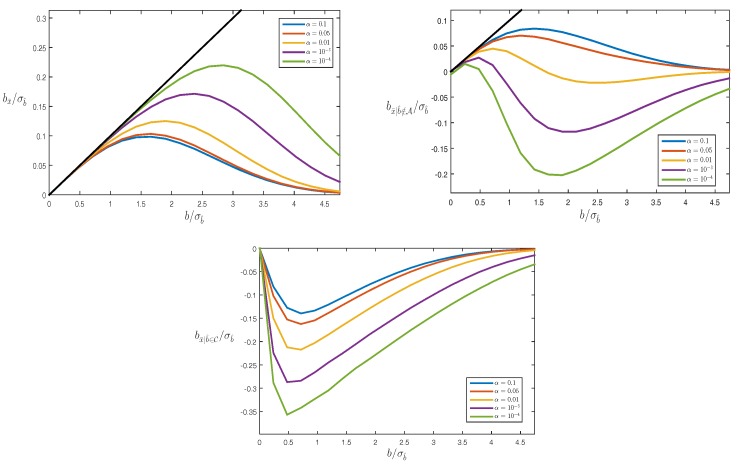
Three types of biases with m=10 for model (29). (top left): unconditional bias $b_{\bar{x}}=E\left(\bar{x}-x|H_{1}\right)$. (top right): correct detection bias $b_{\bar{x}|\mathrm{CD}}=E\left(\bar{x}-x|\mathrm{CD},H_{1}\right)$. (bottom): correct identification bias $b_{\bar{x}|\mathrm{CI}}=E\left(\bar{x}-x|\mathrm{CI},H_{1}\right)$ (C is the correct identification area for $\hat{b}$). The black straight line corresponds with the ’no-testing’ bias $b_{{\hat{x}}_{0}}=\frac{1}{m}b$.

Thus, the data are generated such that the first observation is corrupted with the only outlier *b*. Since $A^{+}=\frac{1}{m}\left[1,\ldots ,1\right]$, the LS-estimators of *x* under $H_{0}$ and $H_{a_{i}}$ are then

(30) $${\hat{x}}_{0}=\frac{1}{m}\sum_{j=1}^{m}y_{j}\;\;\mathrm{and}\;\;{\hat{x}}_{a_{i}}={\hat{x}}_{0}-\frac{1}{m}{\hat{b}}_{i},$$

with ${\hat{b}}_{i}=\frac{m}{m-1}\left(y_{i}-{\hat{x}}_{0}\right)$. The data are generated for different values of *b* and to each such generated data set the above described testing procedure (cf. (24)) is applied. Figure 6 shows the three types of biases $b_{\bar{x}}$, $b_{\bar{x}|\mathrm{CD}}$ and $b_{\bar{x}|\mathrm{CI}}$ that remain after testing. Note that $b_{\bar{x}}$ and $b_{\bar{x}|\mathrm{CI}}$ behave similarly as their one-dimensional counterparts (see Figure 3 and Figure 4). However, the behaviour of $b_{\bar{x}|\mathrm{CD}}$ is different. Here, in contrast to $b_{\bar{x}|\mathrm{CI}}$, the bias still follows in the beginning for small *b* the ’no-testing’ bias $b_{{\hat{x}}_{0}}=\frac{1}{m}b$. This difference in behaviour between $b_{\bar{x}|\mathrm{CD}}$ and $b_{\bar{x}|\mathrm{CI}}$ is due to the multivariate nature of the testing, and it is driven by two multi-dimensional effects. First note, since the multiple testing (24) has used the same critical value as used for the single test in Section 3, that, in actual fact, due to the increase in dimensions, the false alarm probability of the current multiple testing problem is *larger* than α. The probability mass over the acceptance region that needs to be redistributed to account for the correct detection conditioning is therefore smaller than 1-α. Secondly, since in the multivariate case, correct detection admits incorrect identifications, such outcomes make the conditional mean be not larger than *b*, in particular when *b* is still small. This is why the steep decrease, which is present in $b_{\bar{x}|\mathrm{CI}}$, is absent in $b_{\bar{x}|\mathrm{CD}}$.

## 5. Testing Bias in RAIM

**Figure 7:**
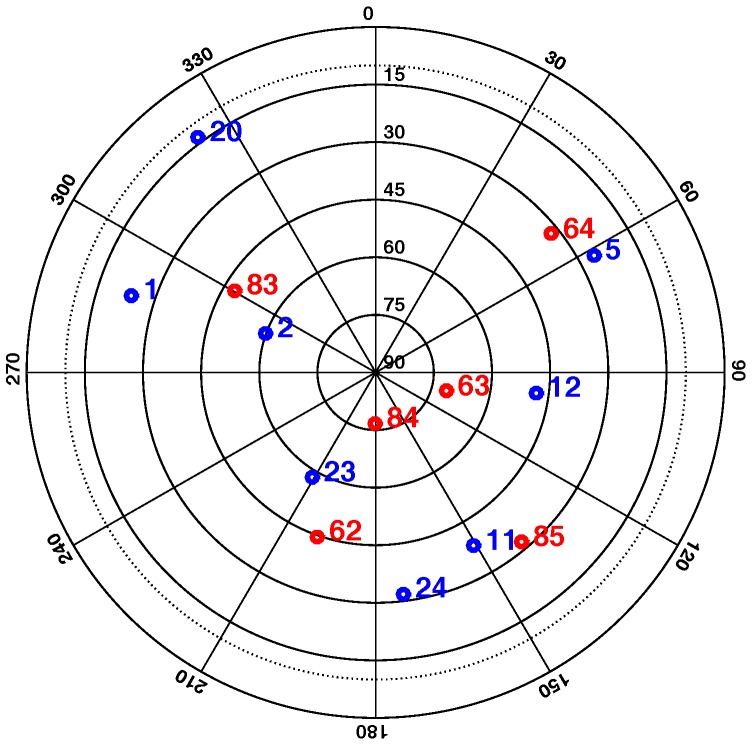
Skyplot for Delft, the Netherlands, showing satellite IDs of nominal GPS (blue) and planned Galileo (red) for the 3rd January 2001 at 2:00 a.m.

As the above revealed and discussed biases are present in any inference procedure that combines estimation with testing, such biases are also present in, for instance, the navigation solution of exclusion-based RAIM. This will be demonstrated by applying the theory of the previous sections to the case of single-receiver pseudorange GNSS positioning. We consider GPS+Galileo with a receiver-satellite geometry as depicted in Figure 7. For a single system, GPS or Galileo, the m×4 design matrix *A* and pseudorange variance matrix $Q_{yy}$ are structured as

(31) $$A=\left[\begin{array}{cc} 1 & u_{1}^{T}\\ \vdots & \vdots \\ 1 & u_{m}^{T} \end{array}\right]\;,\;Q_{yy}=\mathrm{diag}\left(\sigma_{1}^{2},\ldots ,\sigma_{m}^{2}\right),$$

with $u_{i}$ being the *i*th receiver-satellite unit direction vector. The unknown parameter vector $x={\left(dt,dE,dN,dU\right)}^{T}$ consists of the receiver clock offset and the increments to the three position coordinates. Thus, here the ECEF (Earth-Centred, Earth-Fixed) coordinates have already been transformed into the local datum ENU (East-North-Up) coordinates, as these are the coordinates that a practical user in his/her local/national datum will use. Note that if the position coordinates would be known, the GNSS design matrix (31) reduces to that of the example in the previous section. The stochastic model is based on ionosphere-free observations (from dual frequency L1 and L5), with the entries of the diagonal variance matrix in (31) constructed according to Chapter 7 of [21]. For the design matrix of the dual-system GPS+Galileo, an additional column is added to the design matrix above to take care of the inter-system bias or system-specific receiver clock offset.

**Figure 8:**
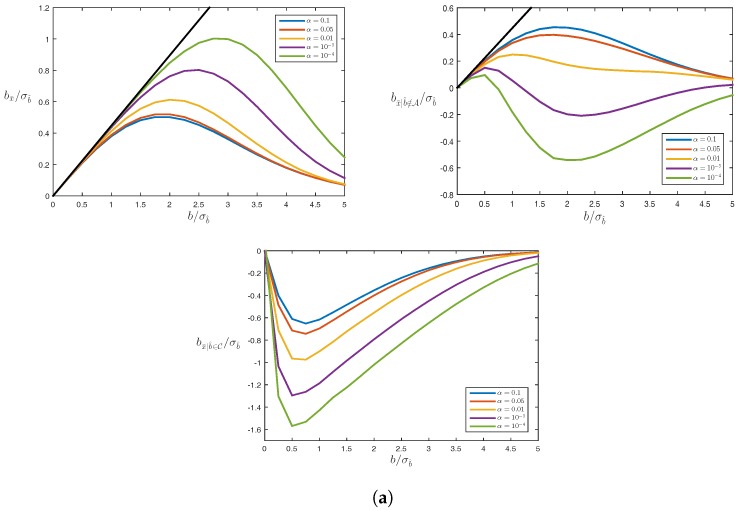

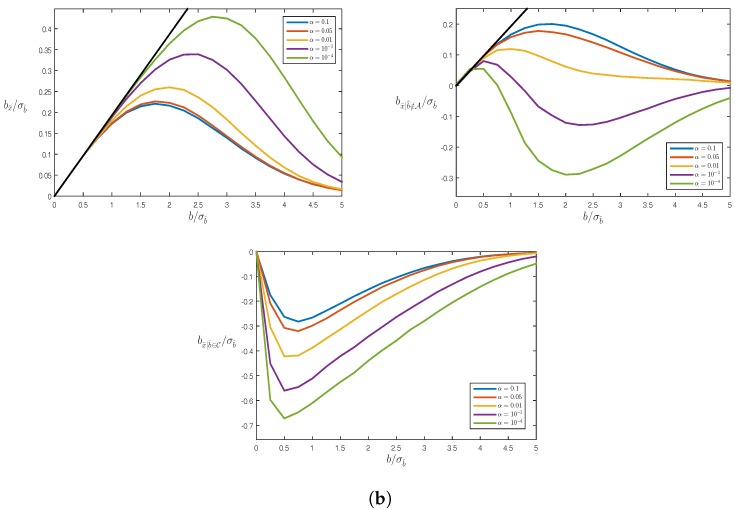
Estimation-testing bias in up-component of GPS+Galileo model (31) due to pseudorange bias in GPS PRN 1 (case (**a**), $\sigma_{\hat{b}}=1.36m$) or in Galileo PRN 64 (case (**b**), $\sigma_{\hat{b}}=1.45m$). Clockwise: unconditional bias $b_{\bar{x}}$, correct detection bias $b_{\bar{x}|\mathrm{CD}}$, and correct identification bias $b_{\bar{x}|\mathrm{CI}}$, with C the correct identification area for $\hat{b}$. The black straight lines correspond with the ’no-testing’ bias $b_{{\hat{x}}_{0}}=A^{+}cb$.

**Figure 9:**
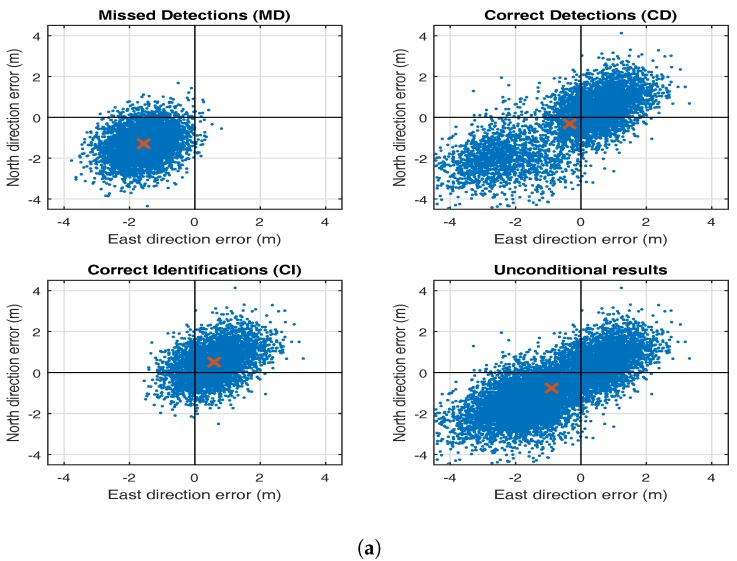

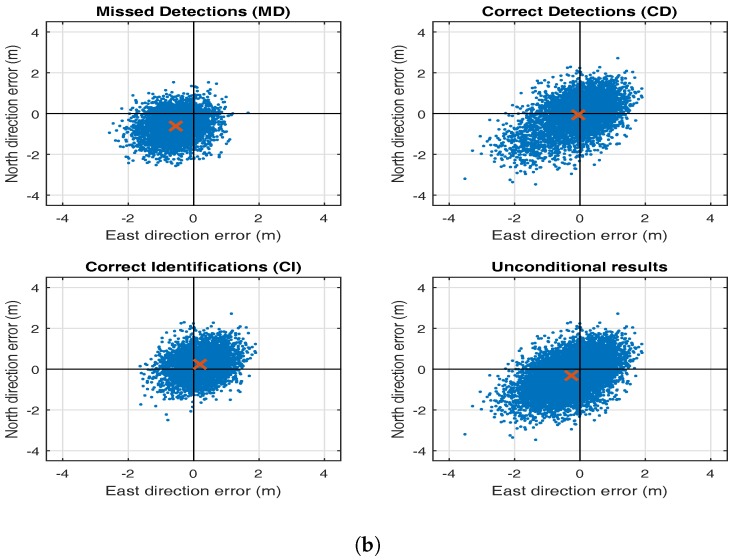
Horizontal position biases, $b_{\bar{x}|MD}$, $b_{\bar{x}|CD}$, $b_{\bar{x}|CI}$ and $b_{\bar{x}}$, for GPS-only pseudorange bias of size $b_{5}=2.5\sigma_{{\hat{b}}_{5}}$ in GPS PRN 5 (**a**) and GPS+Galileo pseudorange bias of size $b_{64}=2.5\sigma_{{\hat{b}}_{64}}$ in Galileo PRN 64 (**b**).

First, we consider the estimation-testing bias in the up-component of the GPS+Galileo model. Figure 8 shows this effect as a result of a pseudorange bias in either PRN 1 (top row) or PRN 64 (bottom row). Note that the behaviour of the three types of biases, $b_{\bar{x}}$, $b_{\bar{x}|\mathrm{CD}}$, and $b_{\bar{x}|\mathrm{CI}}$, is similar to that shown for the previous models. Also note that the impact of the pseudorange outlier in PRN 64 is much smaller than that of the poorer controlled pseudorange outlier in PRN 1 (see Figure 7). To further illustrate the importance of redundancy, Figure 9 shows the horizontal position scatter plots for the GPS-only (top) and GPS + Galileo (bottom) case. The GNSS data were simulated according to (31) but with a single pseudorange outlier included, in PRN 5 for the GPS-only case and in PRN 64 for the GPS + Galileo case (see Figure 7). The same testing procedure as before was applied. The four panels show, for each case, the scatter plots for missed detection (MD), correct detection (CD), correct identification (CI) and the unconditional (UN) case. The scatters of MD and CD together form that of UN, and the scatter of CI is a subset of that of CD. The effect of the increased strength in the GNSS model when using GPS + Galileo instead of GPS-only is clearly visible. In the GPS-only case, as opposed to the GPS + Galileo case, the CD-scatter still contains some quite incorrect identifications due to significant correlations between some of the test statistics. Thus, although the null hypothesis is correctly rejected, these correlations can then still result in incorrect satellite exclusion. Furthermore, even with correct identification (CI), the GPS-only position is in this case off, on average, by more than half a meter. This example has thus illustrated that the a posteriori exclusion of a biased satellite range from the position solution will not remove the bias in the position solution completely and therefore needs to be accounted for in the calculation of the probability of hazardous misleading information.

## 6. Conclusions

In this contribution, we have studied the bias propagation in exclusion-based RAIM. Although statistical testing is intended to remove biases from the data through exclusion, we have shown that biases will *always* remain under the alternative hypothesis, even in the case that such hypothesis is correctly identified. The usage of estimators that are unbiased under their respective hypotheses is therefore no guarantee that the finally computed solution is unbiased as well. We have shown that the presence of such biases in the final solution can be explained by the nonlinearity created by the combination of estimation and testing. The size of these remaining biases depends on the strength of the underlying model, the chosen false alarm rate, and the size and type of the actual input bias. The size of the remaining bias will get smaller with increasing model strength and larger levels of significance. Despite the presence of these biases, the benefit of testing was demonstrated by showing that the remaining bias is always smaller than the biased one otherwise would have been in the absence of testing. However, it was also shown that this is not the case when conditioned solutions are considered. The remaining biases in correctly identified solutions, for instance, can be *larger* than the original input bias. They are small, however, when the input biases are either small or sufficiently large. As the unbiasedness property of the applied estimators is undone when used in combination with testing, one may question whether or not other estimators exist or can be constructed that do a better job in dealing with the discussed bias propagation. In this vein, one may think of relaxing the constraint of using unbiased estimators so that a larger class of estimators can be used to increase flexibility for further improvements of the probability of hazardous misleading information. This is an open question for future research and one that is somewhat similar in spirit to the search for ’integrity optimized estimators’. 

## Figures and Tables

**Table 1:** The mean of the random parameters vectors ${\hat{x}}_{0}$, ${\hat{x}}_{a}$ and $\bar{x}$ under $H_{0}$ and $H_{a}$, respectively.

	$H_{0}$	$H_{a}$
${\hat{x}}_{0}$	$E({\hat{x}}_{0}|H_{0})=x$	$E\left({\hat{x}}_{0}|H_{a}\right)=x+b_{{\hat{x}}_{0}}$
${\hat{x}}_{a}$	$E({\hat{x}}_{a}|H_{0})=x$	$E({\hat{x}}_{a}|H_{a})=x$
$\bar{x}$	$E(\bar{x}|H_{0})=x$	$E\left(\bar{x}|H_{a}\right)=x+b_{\bar{x}}$

**Table 2:** The conditional means of $\bar{x}$ under $H_{0}$ and $H_{a}$.

	$H_{0}$	$H_{a}$
${\bar{x}}_{|\hat{b}\in A}$	$E(\bar{x}|\hat{b}\in A,H_{0})=x$	$E\left(\bar{x}|\hat{b}\in A,H_{a}\right)=x+b_{{\hat{x}}_{0}}$
${\bar{x}}_{|\hat{b}\notin A}$	$E(\bar{x}|\hat{b}\notin A,H_{0})=x$	$E\left(\bar{x}|\hat{b}\notin A,H_{a}\right)=x+b_{\bar{x}|\hat{b}\notin A}$
